# Expanding the Gap: An Updated Look Into Sex Differences in Running Performance

**DOI:** 10.3389/fphys.2021.804149

**Published:** 2022-01-04

**Authors:** Lydia C. Hallam, Fabiano T. Amorim

**Affiliations:** Exercise Physiology Laboratory, Department of Health, Exercise, and Sport Science, University of New Mexico, Albuquerque, NM, United States

**Keywords:** track and field, running, women, performance, sex difference

## Abstract

Males consistently outperform females in athletic endeavors, including running events of standard Olympic distances (100 m to Marathon). The magnitude of this percentage sex difference, i.e., the sex gap, has evolved over time. Two clear trends in sex gap evolution are evident; a narrowing of the gap during the 20th century, followed by a period of stability thereafter. However, an updated perspective on the average sex gap from top 20 athlete performances over the past two decades reveals nuanced trends over time, indicating the sex gap is not fixed. Additionally, the sex gap varies with performance level; the difference in absolute running performance between males and females is lowest for world record/world lead performances and increases in lower-ranked elite athletes. This observation of an increased sex gap with world rank is evident in events 400 m and longer and indicates a lower depth in female competitive standards. Explanations for the sex difference in absolute performance and competition depth include physical (physiological, anatomical, neuromuscular, biomechanical), sociocultural, psychological, and sport-specific factors. It is apparent that females are the disadvantaged sex in sport; therefore, measures should be taken to reduce this discrepancy and enable both sexes to reach their biological performance potential. There is scope to narrow the sex performance gap by addressing inequalities between the sexes in opportunities, provisions, incentives, attitudes/perceptions, research, and media representation.

## Introduction

Competitive sport is one of the clearest examples of high-stakes human endeavor, whereby athletes, coaches and researchers seek to push the limits of human physical performance. Time, money and energy is invested with the aim of athletes obtaining the upper limit of physical capacity and surpassing one another in sporting competitions. Within this context, performance differences between males and females, herein called the “sex gap,” have been studied across sporting events, including athletics, swimming, cycling and rowing (Coast et al., 2004; Cheuvront et al., 2005; Seiler et al., 2007; Thibault et al., 2010; Hunter et al., 2011; Knechtle et al., 2016; Joyner, 2017; Keenan et al., 2018; Millard-Stafford et al., 2018). The best male athletes consistently outperform their female peers, with the magnitude of this sex gap typically ranging between 5 and 17%, depending on the sporting discipline, event duration and competitive standard. For example, the average sex gap for elite level Olympic distance running events is 10.7%, compared to 17.5% for jumps, 8.9% for swimming, and 8.7% for sprint cycling (Thibault et al., 2010). In athletics the sex gap is usually lower for sprints than middle- and long-distances (Coast et al., 2004; Cheuvront et al., 2005; Millard-Stafford et al., 2018). The sex gap within ultra-endurance events is as low as 4.4% in ultra-marathon (Waldvogel et al., 2019). Finally, the sex gap is smaller between elite males and females (Cheuvront et al., 2005; Thibault et al., 2010), compared to sub-elite and recreational runners (Vickers and Vertosick, 2016; Nuell et al., 2019).

The sex gap in sports performance is primarily rooted in biological differences between the sexes, namely in relation to male’s superior skeletal muscle mass, oxidative capacities and lower fat mass (Joyner, 2017). However, there is a range of sociocultural, psychological and sport-specific factors that could explain some of the variance between male and female athletic performance (Deaner, 2006, 2012; Capranica et al., 2013; Donnelly and Donnelly, 2013; Senne, 2016). The relative contribution of these different biological and environmental factors to the sex gap is unclear. Therefore, the aims of this review manuscript are three-fold: (a) provide a summary of the literature on the evolution of the sex gap in running performance, (b) provide an updated analysis on the running sex gap to explore if and how the sex gap has changed in recent years across running events and between performance levels, (c) summarize potential explanations. We chose to focus on the sport of running, specifically flat Olympic distances, because of the availability of result databases and literature on the sex differences in running physiology and performance. Addressing these questions provides insight into the debate surrounding the limits of human athletic performance (Berthelot et al., 2015) and hold practical significance for athletes, coaches and practitioners.

### The Evolution of the Sex Gap: World Records and World Lead Performances

Analyses of world record (WR) performances between 1891 and 2008 reveal two major trends in the sex gap evolution for athletic events: a fast reduction in gap magnitude until the mid-1980s, followed by a period of stability thereafter (Thibault et al., 2010). Within running disciplines 100 m to Marathon distance, the sex gap in WRs decreased from an average of 30% in 1922 to 10.7% at the point of stabilization (∼1985) (Thibault et al., 2010). Table 1 shows the WR sex gaps for specific running events at the established stabilization year [based on Thibault et al.’s analysis (Thibault et al., 2010)], as of 2004 (Cheuvront et al., 2005) and the present day (February 2021). From the mid-1980s to present, there has been an increase in the WR sex gap for all flat Olympic running events, except the 5,000 m and Marathon, where the gap has narrowed slightly (Table 1).

**TABLE 1:** The World Record (WR) times and year for Males and Females, and the WR Sex Gap for Running Events 100 m to Marathon (Cheuvront et al., 2005; Thibault et al., 2010).

Running distance	Male WR time (Year set)^a^	Female WR time (Year set)^a^	WR sex gap (%) during 20^th^ century, taken from Thibault et al. (2010)^b^	WR sex gap (%) as of 2004, taken from Cheuvront et al. (2005)^b^	WR sex gap (%) as of February 2021^b,c^
100 m	9.58 (2009)	10.49 (1988)	6.5	7.3	9.5
200 m	19.19 (2009)	21.34 (1988)	9.2	10.5	11.2
400 m	43.03 (2016)	47.60 (1985)	10.0	10.2	10.6
800 m	1:40.91 (2012)	1:53.28 (1983)	10.4	12.0*^c^*	12.3
1,500 m	3:26.00 (1998)	3:50.07 (2015)	10.6	11.9	11.7
5,000 m	12:35.36 (2020)	14:06.62 (2020)	12.9	14.1	12.1
10,000 m	26:11.00 (2020)	29:17.45 (2016)	10.8	12.1	11.9
Marathon	2:01:39 (2018)	2:14:04 (2019)	10.6	8.4	10.2
Range	1998 – 2020	1983 – 2020	6.5 – 12.9	7.3 – 14.1	9.5 – 12.1

*^a^Time format ss.ms, mm:ss.ms, h:mm:ss.*

*^b^The sex gap (%) is calculated as follows: {[Female WR (s) – Male WR(s)]/Male WR(s)} X 100.*

*^c^Calculated using data from World Athletics public database (https://www.worldathletics.org).*

The trends in the sex gap for WR running performances – rapid decline followed by a period of stability – reflect the changes in the rate at which females were setting WRs compared to males. An analysis of WR progressions reveal that there were significant initial jumps in the rate of female WR advancement which eventually plateaued; whereas, the advancement of male WRs was more gradual throughout the 20th century and into the 21st century (Cheuvront et al., 2005). This reflected the advancement in training, globalization of sport, increase in competitive opportunities and professionalism throughout the 20th century (Joyner, 1993, 2017). Females began competing in sport later than males and were exposed to these changes within a shorter time frame, prompting a drastic fall in female running times unrivaled by that of males (Joyner, 2017). This trend led some to predict that females would eventually outrun males (Whipp, 1992); however, it is apparent that the rate of improvement in female WR performances have leveled, and the sexes now “evolve in parallel” (Thibault et al., 2010). However, the present-day WR sex gaps are slightly wider than they have been in the past (Table 1), and males appear to be breaking WRs more frequently than females (Holden, 2004). As such, males may be improving at a greater rate than females at the world class level.

Considering many female WRs have not been surpassed since the 1980s (Table 1), it is also insightful to consider progression in the annual world lead (WL) performances for both sexes. Sparling et al. found the sex gaps for WL 1,500 m and Marathon performances were relatively stable between 1980 and 1996, averaging at 11.1% and 11.2%, respectively (Sparling et al., 1998). This indicates that the magnitude and trends in the WL sex gap are similar to that of WR performances (Cheuvront et al., 2005).

The literature on the sex gap evolution in running often focuses on changes in WR or WL performances (Sparling et al., 1998; Cheuvront et al., 2005; Thibault et al., 2010; Sandbakk et al., 2017). Whilst these analyses give insight to the upper limits of human performance (Coast et al., 2004), we should be cautious when drawing conclusions about general trends in the sex gap based on these rare and extraordinary individual performances (Capranica et al., 2013). These studies use a single data point per year (i.e., percent difference between the #1 ranked male and female); therefore, “anomalous” performances could skew the trends and subsequent conclusions drawn. This is of concern within the context of doping scandals and whether some WRs were achieved by athletes taking performance-enhancing drugs. World Athletics have considered a proposal to erase all WRs set before 2005, the year when blood and urine samples were first stored for future testing (Edwards et al., 2018). If this reset took place, the sex gap for some events would be impacted quite drastically, for example the women’s 100 m WR gap would increase from 9.5 to 11.1%. This brings into the question the relevance of studying WRs to understand the sex gap evolution, since such records may not always be reliable or trustworthy markers of the natural limits of human performance.

### The Evolution of the Sex Gap: Elite Performances

Whilst coaches, athletes, scientists and sports teams are looking to push the limits of human athletic performance by achieving WR times, the world of high-performance sport is also concerned with advancing competitive standards within the elite category as a whole. Looking beyond WRs/WLs and taking the average sex gap between a small group of the top ranked male and female runners will expand the pool of data analyzed each year. This can provide a more representative picture of trends in the sex gap evolution for world class runners and allow detection of changes not reflected in WRs/WLs alone (Seiler et al., 2007; Capranica et al., 2013; Sandbakk et al., 2017). Such an approach would also help account for the confounding effect of performances achieved by doping and/or anomalies in sport.

A few studies have adopted this approach (Seiler et al., 2007; Thibault et al., 2010; Millard-Stafford et al., 2018). Millard-Stafford et al. compared the top 8 male and female performers at the U.S. Olympic track and field Trials and found the average sex gap for running events was 12.1% in 2016, which had been stable since 1980 (Millard-Stafford et al., 2018). The gap had decreased from 17.3% in 1972 following the instigation of Title IX, a federal law which legislated equal opportunities for females in the United States education system, including within sport. Additionally, Thibault et al. examined the sex gap evolution using the top 10 male and female running performances in athletics worldwide (Thibault et al., 2010). The average gap decreased from 25.3% in the early 20th century to 11.2% in 1984, after which followed a stabilization period (Thibault et al., 2010). Seiler et al. studied the sex gap between the top 6 finishers in male and female World Championship and Olympic Games finals for sprint events. The average sex gap for the 100 m, 200 m and 400 m events decreased between the 1950s and 1980s to reach an average low of 9.8%, before increasing to 11.2% by 2005 (Seiler et al., 2007).

Moreover, the patterns in the sex gap evolution are similar when studying the elite pool and WR/WL performances – rapid decline until the 1980s then stabilizing (Thibault et al., 2010). However, as demonstrated by Seiler et al., looking at the top 6 annual performances at major championships allows sensitivity to detect the widening of the sex gap over a shorter time frame in more recent years (Seiler et al., 2007). It is unclear whether a similar change has occurred in middle- and long-distance running events, or if there are trends that have occurred in the sex gap in the last decade. Further, the above studies are insightful but limited because they only consider performances from a single nation/at a single event [United States Olympic Trials (Millard-Stafford et al., 2018)], or do not include the most recent years’ data on sex gaps [data until 2008 (Thibault et al., 2010), data until 2005 (Seiler et al., 2007)].

### An Update on the Sex Gap in Running Performance

#### Sex Gap Trends for Top 20 Performances Over the Last 20 Years

Understanding trends in the sex gap in the modern athletic era will give insight into the current competitive standard of male and female running and how the sexes perform relative to one another. This should guide discussion around explanations for the sex gap and how to advance athletic performance in both sexes.

We conducted an up-to-date analysis on the sex gap between elite male and female runners using the annual top 20 world best performances over the past two decades. Data was extracted from the World Athletics public database (^1^ Season Top Lists, date extracted: 02/01/2021), with the top 20 best by athlete marks selected for male and female Olympic running distances (flat events: 100 m, 200 m, 400 m, 800 m, 1,500 m, 5,000 m, 10,000 m, Marathon) each year between 2001 and 2020. For each event and each year, a pairwise comparison was made between the nth ranked male and female performance time (seconds), using the following equation:


$$sexgap\left(\%\right)=\frac{\left\{\left[\frac{F\,e\,m\,a\,l\,e_{n}\,\left(s\,e\,c\right)-M\,a\,l\,e_{n}\,\left(s\,e\,c\right)}{M\,a\,l\,e_{n}\,\left(s\,e\,c\right)}\right]\times 100\right\}}{20}$$

For each event each year, the average sex gap across all 20 ranks was calculated as follows:


Top 20sexgap(%)={∑[F⁢e⁢m⁢a⁢l⁢en⁢(s⁢e⁢c)-M⁢a⁢l⁢en⁢(s⁢e⁢c)]M⁢a⁢l⁢en⁢(s⁢e⁢c)}×100

As per Seiler et al. (2007), we did not analyze the data with inferential statistics, considering data points were compiled from multiple individuals across multiple years. The sex gap (%) in performance time for each pairwise rank was plotted against historical time (year) using GraphPad Prism 8 (San Diego, CA) for each running event. Two regression lines were fitted to the data: a linear regression (y = ax + b) and a second order polynomial regression (y = ax2 + bx + c). If the R2 value was improved by at least 0.02 when using the second order polynomial regression, this was used as the line of best fit, otherwise the linear regression was used (Seiler et al., 2007).

**FIGURE 1:**
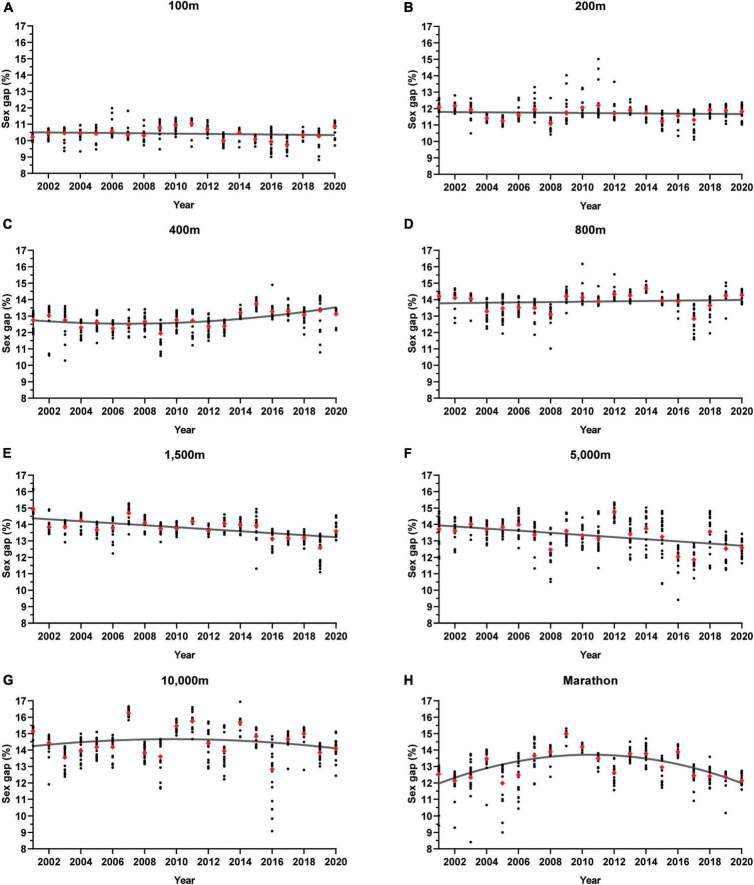
The Sex Gap Over Time in Running Events 100 m to Marathon between 2001 and 2020. The sex gap % is calculated as the percent difference between male and female running times. The pairwise sex gaps for each top 20 ranked individuals (annually, worldwide) are plotted as black circles and the average top 20 sex gap for each year is plotted as a red diamond. Regression lines are fitted as the line of best fit. **(A)** 100 m, **(B)** 200 m, **(C)** 400 m, **(D)** 800 m, **(E)** 1,500 m, **(F)** 5,000 m, and **(G)** Marathon.

As inferred from the regression lines plotted to the data (Figures 1A–H), the sex gap for top 20 performers over the past two decades has been relatively stable for the 100 m, 200 m and 800 m events, has increased slightly for the 400 m, has decreased slightly for the 1,500 m and 5,000 m, and has fluctuated for the 10,000 m and Marathon. The current trends in the top 20 sex gap for sprints (100 m, 200 m, 400 m), middle-distances (800 m, 1,500 m) and long-distances (5,000 m, 10,000 m, Marathon) will be discussed in relation to the WR sex gaps and the literature below.

##### Sprints

In accordance with other studies (Coast et al., 2004; Millard-Stafford et al., 2018), the present analysis shows that the top 20 sex gap for sprinters (specifically 100 m and 200 m) is consistently lower than middle- and long-distance runners over the years (Figure 1). The average top 20 sex gap for the 100 m ranges from 9.75 to 11% between 2001 and 2020 (Figure 1A), consistently higher than the current 100 m WR sex gap of 9.5% (Table 1). For the 200 m, the average top 20 gap is, on occasion, similar to the WR sex gap of 11.2%, but most years it exceeds 11.5%. The 400 m top 20 sex gap has exceeded 12% every year in the past two decades, whereas the WR sex gap is 10.6%.

**FIGURE 2:**
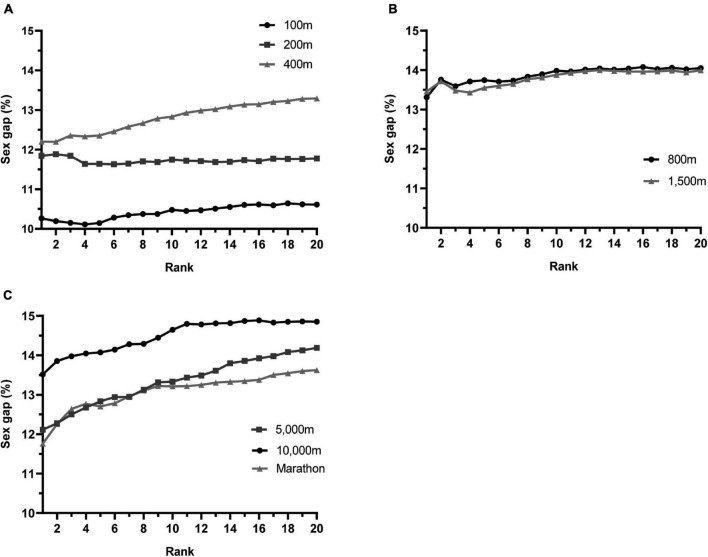
The Sex Gap and Performance Level in Running Events 100 m to Marathon. The sex gap % is calculated as the percent difference between male and female running times, averaged over a 20-year period (2001 – 2020) for each worldwide rank position (1 through 20) for **(A)** sprint events, **(B)** middle-distances, **(C)** long-distances.

Seiler et al. observed a narrower sex gap in the sprints in the 1980s compared to the 2000 – 2005 period (Seiler et al., 2007). This may have been an artifact of doping prior to the initiation of randomized drug testing in 1989; the gains that male athletes receive from performance-enhancing drugs may be smaller than that of females due to the male’s pre-existing high levels of circulating testosterone (Seiler et al., 2007). It appears that since the early 2000 s, the average sex gap has not changed drastically for the 100 m or 200 m and may be increasing very slightly in the 400 m (Figure 2). This implies that the confounding effect of performance-enhancing drugs are minimized, allowing a more accurate assessment of the limits of male and female performance.

##### Middle-Distance

The average top 20 sex gaps for the 800 m and 1,500 m exceeded 13% in all but one year, and the present WR sex gaps for these events are 12.3% and 11.7%, respectively (Table 1). Similarly, Thibault et al. established the stable sex gap for the top 10 800 m and 1,500 m performances at 11.3% and 12.3%, compared to 10.4% and 10.6% for WR performances, respectively (Thibault et al., 2010).

The sex gap in elite 1,500 m appears to have decreased in recent years (Figure 1E), an insight which is not apparent based on earlier studies (Sparling et al., 1998; Thibault et al., 2010). Whilst speculative, the narrowing of the gap could reflect rising standards of elite female 1,500 m, particularly amongst British and American runners. Between 2005 and 2008, either no athletes or a single female athlete was represented by these countries on the top 20 world rankings. In comparison, four American and four British female runners were ranked in the top 20 in 2019. Whilst the WR sex gap has not narrowed significantly since 2004 (Table 1), the top 20 sex gap for 1,500 m is ∼1% lower, and it will be of interest to see if this trend continues.

The overall trend in the 800 m sex gap is stable based on the fitted regression line (Figure 1D), which is consistent with the findings of Thibault et al. (2010). However, visual inspection of the graph reveals nuances in 800 m sex gap evolution; there is a cyclical trend in the sex gap – decreasing between 2001 and 2008, then increasing to 2013, decreasing to 2017 before increasing in the subsequent 3 years.

##### Long-Distance

On average, the top 20 sex gap was highest of all running events for the 10,000 m. There was no systematic increase in the sex gap with increasing event distance as suggested elsewhere (Coast et al., 2004), since the 5,000 m and Marathon gaps were typically lower than that of the middle-distances (Figure 1).

The sex gap has narrowed in recent years for the 5,000 m (Figure 1F); at the start of the era (early 2000 s), the sex gap appeared relatively stable around 13.8%, yet has been ∼1 – 2% lower in comparison in the last 5 years (with the exception of 2018). Alongside this, there appears to be a concomitant narrowing of the sex gap in the 5,000 m WR; in 2004 this was 14% (Table 1), compared to the current gap of 12%.

As seen in Figure 1G, there is large annual variability and fluctuations in the sex gap for the 10,000 m. This is not particularly evident in the sprints and middle-distances, where year-on-year variations in the sex gap are smaller and more cyclical. The average top 20 sex gap for the 10,000 m was lowest at 12.8% in 2016 and highest at 16.2% in 2007, and consistently higher than the current WR sex gap of 11.9% (Table 1).

The Marathon event also displays similar variability in the sex gap, with an overall inverted-U shape trend, increasing for the first decade then decreasing (Figure 1H). The average sex gap always exceeded 12%, compared to the current Marathon WR sex gap of 10.2%. Moreover, Hunter et al. found that, between the late 1990’s and 2009, the average sex gap for the top 5 runners at major world class marathon races differed significantly over the years; although, there was no systematic trend to indicate the sex gap narrowing or widening (Hunter et al., 2011).

These observations indicate that, although there may be overall stability in the sex gap for the longer distances, annual changes occur in how females perform relative to their male peers, and vice versa. Whilst it is currently unclear why such variations exist it could be inferred that multiple underlying variables are involved.

#### Changes With Performance Level Over Top 20 Ranks

To further understand the relationship between the sex gap and performance level, we averaged the sex gap across the 20-year period (2001 – 2020) for each rank position (1st to 20th) using the pairwise sex gaps for the top 20 performers across Olympic running events. For each event (100 m to Marathon), we plotted the averaged sex gap against rank position.

As illustrated in Figure 2, the overall trend for most running events is an increase in the sex gap as the rank increases from the 1st to 20th athlete. For the sprint events (Figure 2A), the increase in sex gap with rank is most clearly seen for the 400 m. For the 100 m, the sex gap decreases from 1st to 4th ranked individuals, increases to the 15th rank, then plateaus. For the 200 m, the gap is highest for the first three ranked individuals and is relatively similar between 4th and 20th ranks. For the middle-distances (Figure 2B), changes in sex gap with rank position are similar; there is a clear trend toward a gradual expanding of the sex gap from 1st to 13th rank, after which the gap is relatively stable at ∼14% for higher ranks. There is a linear trend of increase in the 5,000 m sex gap from 1st to 20th ranked athlete. For the 10,000 m and Marathon, there is a relatively large jump in the sex gap from 1st to 4th rank, and a more gradual expansion of the gap with each rank position thereafter. The difference in average sex gap between 1st and 20th ranks is smallest for distances 100 m to 1,500 m (<1%), and larger for 5,000 m to Marathon (1.3 – 2.1%).

The widening of the sex gap with rank position or finishing place in elite level runners is evident in other studies (Sparling et al., 1998; Hunter et al., 2011, 2015; Hunter and Stevens, 2013; Senefeld et al., 2015). The sex gap increases from the WL performers to the 100th ranked athletes in the 1,500 m (Sparling et al., 1998) and Marathon (Sparling et al., 1998; Hunter et al., 2015), from the 1st place to 5th place finisher (Hunter et al., 2011) and 1st to 10th place finisher (Hunter and Stevens, 2013; Senefeld et al., 2015) in world class marathon races. This is due to a greater relative drop off in performance time with increasing rank/position for female athletes (Hunter and Stevens, 2013).

Collectively, these findings indicate that male athletes are relatively faster runners and closer to the sex specific WR/WL marks than females, and that the competitive pool of male runners is more homogenous. This is more apparent in middle- and long-distance running than in short sprints, where the sex gap is not noticeably different between 1st and 20th world class ranks (Figure 2). Nonetheless, there is evidence of a greater drop off in female performance at lower performance levels for sprinters; the sex gap for sub-elite sprinters is 15% across 80 m (Nuell et al., 2019), which is higher than the 100 m sex gap for WR and elite performances. Moreover, there is a sex gap in relative performance and depth of competition within running.

## Explanations

Underpinning differences in running performance between males and females are a range of potential physical (physiological, anatomical, neuromuscular, biomechanical), sociocultural, psychological and sport-specific factors. The role each variable contributes to the sex gap may vary between event disciplines, across historical time, across cultures, and with performance level. Many assert that the gap in running today is explained purely by biological sex differences (Cheuvront et al., 2005; Thibault et al., 2010; Millard-Stafford et al., 2018). However, there are fluctuations and small changes in the sex gap for WRs (Table 1) and elite level running (Figure 1), which warrant further investigation into other explanatory factors. This has significance to the discussion of whether runners, both male and female, have reached the limits of human performance, or whether advancements in technology, training, opportunities and talent identification could see notable jumps in one or both of the sexes (Berthelot et al., 2015). The following section will discuss the biological and environmental explanations for the sex gap in running performance.

### Physiological, Anatomical, Neuromuscular and Biomechanical Explanations

#### Sprints

The sex differences in muscle anatomy and physiology are significant in explaining the performance gap in sprint running. Male runners have superior muscle volumes than females (Nuell et al., 2019), due to longer muscle segment sizes and ∼25 – 40% more skeletal muscle mass (Sandbakk et al., 2017). The latter is due to male’s larger muscle fiber cross sectional areas, as opposed to greater fiber numbers (Miller et al., 1993; Jaworowski et al., 2002). Since muscle cross sectional area is closely related to the force producing capacity of the muscle (Miller et al., 1993), males are naturally stronger and more powerful than females, contributing to their advantage in sprinting (Sandbakk et al., 2017). Additionally, males have superior anaerobic metabolic power than females (Sandbakk et al., 2017), potentially due to a larger relative area of fast twitch muscle fibers which have large glycolytic capacity (Jaworowski et al., 2002) and higher peak tension (Schiaffino and Reggiani, 2012). These differences in muscle morphology and function arise during puberty and are underpinned by the increase in circulating testosterone in males (Handelsman, 2017).

Biomechanical and neuromuscular factors are also pertinent to this discussion for sprinters. It is apparent that sprint speed is limited by the ability to apply large ground reaction forces over short contact times, as opposed to the anaerobic energy supply (Bundle and Weyand, 2012). Male sprinters possess anthropometric, structural and mechanical properties that favor their ability to produce horizontal ground forces at high speeds (Nuell et al., 2019). These include their larger skeletons, longer legs and therefore longer stride lengths, higher center of gravity and greater muscle and tendon stiffness (Blackburn et al., 2006; Slawinski et al., 2015; Nuell et al., 2019). These variables contribute to sex differences in mechanical sprint properties in both elite (Slawinski et al., 2015) and sub-elite sprinters (Nuell et al., 2019). Males obtain greater maximal force, velocity and power, have longer acceleration phases and shorter deceleration phases than females.

Moreover, if sex differences in body composition are critical in explaining the male advantage in running performance, one may expect the sex gap to be greater in disciplines that rely predominantly on muscular strength and power. However, this is not the case; the sex gap for short sprint events (100 m and 200 m) are typically smaller than distance events (Coast et al., 2004; Sandbakk et al., 2017; Millard-Stafford et al., 2018) (Figures 1, 2 and Table 1). A potential reason for this is that female sprinters have somewhat of an advantage due to their relatively smaller upper-body mass, meaning there is less inertia to overcome when accelerating (Sandbakk et al., 2017).

#### Middle-Distance

Middle-distance running, particularly the 800 m, is an interesting and challenging field for sports physiologists to study because it represents the “middle ground” between aerobic and anaerobic energy domains (Duffield and Dawson, 2003). The estimated anaerobic energy contribution to 800 m running is higher in males than females (Duffield and Dawson, 2003; Duffield et al., 2005) which could be related to sex differences in muscle fiber type distribution, with male middle-distance runners displaying a greater proportion of fast twitch fibers (Costill et al., 1976, 1979).

Furthermore, it is recognized that elite male 800 m runners possess biomechanical and neuromuscular abilities that underpin fast maximal sprinting speed (MSS), which is a requirement for competitive success (Sandford et al., 2018, 2019). Sandford et al. found that elite male 800 m runners have a large anaerobic speed reserve (ASR) – the difference between MSS and the velocity at VO_2_max (Sandford et al., 2018). Possessing a large ASR may be advantageous because it signifies the athlete has a large “race pace” speed bandwidth in which they can adjust velocity to produce mid-race surges and end-kicks, as well as the force application abilities to execute very fast starting velocities (Sandford et al., 2019). The role of the ASR in female middle-distance running is an unstudied area, and it is unclear whether female 800 m runners possess these characteristics that are critical for success in male running.

Hence, the physiological, biomechanical and neuromuscular profiles of a typical female middle-distance runner could mean they have greater potential to excel at longer aerobic-based events than the 800 m (Duffield and Dawson, 2003; Duffield et al., 2005). If this is true, then you may expect the sex gap to be greater in the 800 m than other middle- and long-distances. This was not clear from the top 20 sex gap analysis; however, we did see a narrowing of the gap over time for the 1,500 m and 5,000 m and this trend was absent for the 800 m (Figure 1). Perhaps females are “training smarter” in these longer events, leading to greater relative improvement compared to males. There may be scope for female 800 m runners to narrow the sex gap by tapping into the ASR domain, which is evidently a successful approach for male middle-distance runners (Sandford et al., 2018). There is a male sex bias in middle-distance running research (Mpholwane, 2007) which means that coaches tend to train female athletes using strategies that have been validated in males, but do not take into consideration aforementioned sex differences. Hence, more research is needed using female runners to ensure that training strategies will enable them to reach their biological potential.

It is also important to consider the large diversity in physiological and mechanical profiles of middle-distance runners (Sandford and Stellingwerff, 2019). Sandford identified speed-based and endurance-based subtypes in male 800 m runners, and asserted that both are capable of executing successful performances through adopting tactics/pacing that favor their underlying physiologies and mechanics (Sandford et al., 2018). We have observed similar subtype categorizations in female 800 m runners (unpublished data). Hence, coaches and athletes should be aware of their unique profile and use this information to advise training and competition practices.

#### Long-Distance

Over long-distances, the variance between sexes is primarily related to differences in maximal oxygen consumption (VO_2_max). Elite male marathon runners have relative VO_2_max values that are ∼10% higher than their female peers (Pate and O’Neill, 2007). This is because males have less fat mass and greater skeletal muscle mass, the most metabolically active tissue in the body, than do females (Wang et al., 2010). Additional physiological variables likely contribute to the superior cardiorespiratory capacity in males. Males have greater red blood cell and hemoglobin concentrations than females, therefore a higher blood oxygen carrying capacity (Cureton et al., 1986; Murphy, 2014). Additionally, males have larger hearts and lungs relative to body size (Purkiss and Huckell, 1997; Harms et al., 1998; LoMauro and Aliverti, 2018), as well as larger cardiovascular preload and stroke volumes (Huxley, 2007; Wheatley et al., 2014). Since VO_2_max represents the upper limits in aerobic capacity and maximal rate of oxidative ATP production (Bassett and Howley, 2000), it is clearly a key determinant of endurance running performance difference between the sexes (Cheuvront et al., 2005; Joyner, 2017).

Physiological differences related to thermoregulation (Cheuvront et al., 2005) and metabolism (Tarnopolsky et al., 1990; Carter et al., 2001) could explain some of the sex variance in long-distance running performance; although, their role is less clear. Females have greater reliance on lipid oxidation during prolonged submaximal exercise than males (Tarnopolsky et al., 1990; Carter et al., 2001), and this may give them an advantage over marathon distances due to glycogen sparing and delaying fatigue (Sandbakk et al., 2017). In support, the average top 20 sex gap for the Marathon is typically lower than other long- and middle-distance events (Figure 1). However, this advantage in fuel regulation may be negated in Marathon racing, where both sexes use regular carbohydrate feeding which will reduce reliance on lipid metabolism (Coast et al., 2004). There is no conclusive evidence for sex differences in mitochondrial capacity, lactate threshold or running economy (Joyner, 2017), which are also key determinants of endurance running performance (Bassett and Howley, 2000).

### Gender Equality in Participation and Opportunity

The sociocultural conditions hypothesis states that sex differences in opportunities and participation explain variance between male and female sports performance (above that which is rooted in biological differences) and the lower depth of female competition (Hunter et al., 2015; Keenan et al., 2018). Female participation in athletics has increased drastically since the first modern Olympic games in 1896, where not a single female competed. Legislative changes throughout the 20th century meant that females were permitted to compete in events from which they were previously banned, for example in the Olympic 1,500 m in 1972 and the Marathon in 1982 (Cheuvront et al., 2005). Three milestones were achieved at the London 2012 Olympic Games: 44.3% of participants were females, the highest percentage of any Summer Olympic Games; females competed in every event; all nations were represented by female athletes (Donnelly and Donnelly, 2013). Title IX legislation has promoted equal opportunities for females to train and compete at the U.S. collegiate level and has equalized the financial aid awarded to males and females within an institution (Senne, 2016).

Despite the progress made in legislation, policies and participation, sex inequality still pervades sport and sociocultural factors are likely to influence the sex gap today (Capranica et al., 2013). There is a discrepancy in funding and financial incentives, social support provided by governing bodies and sporting federations, and media representation between male and female athletes (Capranica et al., 2013; Donnelly and Donnelly, 2013; Senne, 2016). Alongside this, stereotypes and perceptions remain a barrier to female athletes; sport is viewed by many as a masculinized domain and despite opportunities, females are still less likely to participate than males (Senne, 2016).

Some have argued against the sociocultural conditions hypothesis as an explanation for the sex gap in absolute performance and competition depth. The frequency at which females set WRs in athletics decreased between 1980s and 2008, despite growing numbers of female participants at the Olympics, indicating that participation is not a limiting factor for the progression of female performance (Thibault et al., 2010). However, this is an incomplete argument; despite roughly equal numbers of male and female Olympic competitors, females are still underrepresented at the sub-elite and grassroots level (Deaner, 2006; Capranica et al., 2013; Senne, 2016). This could be the source of the sex gap in competitive depth at higher levels of competition. Additionally, whilst female athletes in countries such as the United States benefit from legislation promoting equal opportunities and participation in sport, this is not universal (Capranica et al., 2013).

Furthermore, Keenan et al. (2018) found that female collegiate rowers improved more than their male peers between 1997 and 2016, narrowing the sex gap in absolute performance and competition depth. These changes coincided with an increase in female, but not male, participation. Additionally, lower female participation is associated with an increase in the sex gap in Marathon running (Hunter and Stevens, 2013). These examples demonstrate that participation is an important factor related to changes in female performance and the sex gap evolution, corroborating the sociocultural conditions hypothesis.

The expanding of the sex gap with rank position, an indicator of lower depth in the female field, is more apparent in the longer distances (Figure 2). In the 5,000 m, 10,000 m and Marathon, there is a larger increase in the sex gap with rank, compared to the 100 m and 200 m (and to a lesser extent 800 m and 1,500 m) where the changes between 1st and 20th ranks are small. This could be because females have been competing over long-distances for a shorter time (the first Olympic Marathon for females took place in 1984) resulting in a time lag effect. We may see an improvement in depth of long-distance running over time as more females have the opportunity to train and compete in the sport.

### Competitiveness and Psychology

The evolved predispositions hypothesis argues that there are innate and evolved sex differences in competitiveness and motivation that explain the sex gap in competition depth (Deaner, 2006, 2012; Keenan et al., 2018). Deaner (2012) argues that males have a predisposition for enduring competitiveness, meaning they are attracted to performance-based environments where they display and compete for status. As such, males are more likely to be dedicated to the training regimens that are required for success in sport; this drives up the standard and depth of male competition. In support, Deaner found that in the United States more male high school, collegiate and professional track runners ran relatively fast compared to the sex-specific world class standards than did their females counterparts (Deaner, 2006).

When investigating potential sex differences in the psychological disposition toward competitive environments, it is useful to analyze how male and female runners execute performance, i.e., their pacing strategy. Pacing can be described as the distribution of metabolic resources over the course of a race (Abbiss and Laursen, 2008). Female runners typically display more even pacing profiles across middle- and long-distances at the elite (Hanley, 2016; Filipas et al., 2018) and recreational level (March et al., 2011; Trubee et al., 2014; Deaner et al., 2015, 2016). Male runners are more likely to adopt risky and ambitious pacing strategies – starting too fast and slowing significantly in the second half of the race – which could reflect an overconfidence in their abilities. This is consistent with the evolved predispositions hypothesis. Conversely, Hanley and Hettinga (2018) found that both sexes displayed “ego-oriented behavior” in major championship 800 m and 1,500 m races. The eventual gold medalists ran to win during qualifying rounds, as opposed to doing just enough to qualify and preserve energy reserves for the final. Other studies demonstrate similarities in race strategies between the sexes, particularly amongst medalists (Hanley et al., 2019; Hettinga et al., 2019). This indicates elite female runners display equally competitive or risky racing behaviors as males; however, perhaps relatively fewer females have such a disposition at lower competitive levels.

One could integrate aspects of both the enduring competitiveness and sociocultural conditions hypotheses to explain why fewer females engage in competitive running. Perhaps males possess a greater competitive drive to pursue sporting success from a young age because they are primed by their sociocultural environment to desire/expect this. Likewise, females may anticipate fewer opportunities, less acceptance and less support, so have less motivation to engage in sport.

## Practical Applications

Why should we be concerned with how the sex gap in elite runners is evolving or the underlying explanations? The purpose of this discussion is not to create a “battle of the sexes” (Tanaka, 2002), since males and females are not in direct competition with each other but, rather, with members of their own sex. This discussion should instead be approached with the view of seeing both male and female athletes maximize their biological potential and push the limits of human performance. If this is achieved by both sexes, the magnitude of the gap is insignificant. However, if it is apparent that one sex has more opportunities, support, incentives and provisions to reach their performance potential, we are faced with an inequality that should be challenged. As argued in this review, females historically and presently are the disadvantaged sex within sport.

It appears that there is scope to narrow the sex gap for elite runners, particularly in relation to competition depth. Sex gaps as low as 10 – 11% are biologically possible for the best male and female runners (Table 1), but we consistently observe increasing sex gaps at lower performance levels (Figure 2). To address the sex gap in competitive depth, governing bodies and sporting federations should implement strategies to expand the talent pool from which the world’s best athletes are drawn. This should focus on increasing female participation in grassroots sport, improving talent identification schemes to recognize young female athletic talent, and retaining these athletes in the transition from junior to senior level competition. Media campaigns should be used to challenge the stereotypical view that sport is a masculine domain and grow commercial interest in female sport. More research in female runners is needed so that coaches and practitioners better understand the unique training responses, race demands, physiologies and mechanics of their female athletes. Finally, financial provisions and incentives should be equalized between the sexes, particularly in countries where there are fewer policies and legislations in place that support the female athlete.

A limitation of the present study is the lack of use of inferential statistics. Although the data set used is representative of the population studied (top 20 best male and female runners between 2001 and 2020), some runners appeared in different years and events multiple times. These multiple appearances for the same runners violate the independence assumption where each data element must be selected independently of data previously selected. Another limitation is the analyses of a relatively short time frame (20 years) and the observed small fluctuations in the sex gap over the last 20 years. Therefore, we recommended here, a word of caution, in regard to meaningful and practical significance.

## Conclusion

It is undeniable that the drastic narrowing of the sex gap during the 20th century has leveled off, and the current data indicates that performance will not be completely equalized between the sexes, at least for Olympic running distances (Cheuvront et al., 2005; Thibault et al., 2010; Millard-Stafford et al., 2018). However, the assertions that the sex gap is now fixed and explained entirely by biological sex differences is a limited explanation. The sex gap in athletic performance is not a stable entity; elite male and female runners are constantly evolving in relation to their same-sex competitors and to one another. In the last two decades, the top 20 sex gap has widened slightly for the 400 m, narrowed slightly for the 1,500 m and 5,000 m, and had fluctuated for the 10,000 m and Marathon (Figure 1). This could be due to a range of integrated biological and environmental factors; shifts in training strategies, technologies, research, event demands, opportunities, participation, provisions, legislation and perceptions can all play a role in performance progression in runners. When one sex experiences differential advantages from these factors, this is likely to be reflected in a change – narrowing or widening – in the sex gap. The present review demonstrates that there is not just a sex gap in absolute performance, but also in competition depth. The sex gap increases with rank position, i.e., at a lower performance level, in events 400 m and longer (Figure 2). This suggests there are more male runners that are closer to the sex-specific world class standard than females, and that the male competitive field is more homogenous. Seeking to rectify this gap will require a multidisciplinary approach to the address the sex biases that pervade sport and research.

## Author Contributions

LH conducted the literature review and data analysis. LH and FA contributed to the conceptualization of ideas, drafting, and critically revising the work. Both authors contributed to the article and approved the submitted version.

## Conflict of Interest

The authors declare that the research was conducted in the absence of any commercial or financial relationships that could be construed as a potential conflict of interest.

## Publisher’s Note

All claims expressed in this article are solely those of the authors and do not necessarily represent those of their affiliated organizations, or those of the publisher, the editors and the reviewers. Any product that may be evaluated in this article, or claim that may be made by its manufacturer, is not guaranteed or endorsed by the publisher.

## Footnotes

1
https://www.worldathletics.org
